# Editorial for the Special Issue “Targeting Tumor Microenvironment for Cancer Therapy, 3rd Edition”

**DOI:** 10.3390/cimb48020155

**Published:** 2026-01-30

**Authors:** Cheng-Che Lee, Ming-Wei Lin

**Affiliations:** 1 Department of Occupational Therapy, I-Shou University, Kaohsiung 82445, Taiwan; chengche@isu.edu.tw; 2 School of Medicine, I-Shou University, Kaohsiung 82445, Taiwan; 3 Department of Medical Research, E-Da Cancer Hospital, I-Shou University, Kaohsiung 82445, Taiwan; 4 Department of Nursing, College of Medicine, I-Shou University, Kaohsiung 82445, Taiwan

The tumor microenvironment (TME) is a highly dynamic and heterogeneous ecosystem composed of cancer cells, stromal components, immune cells, and extracellular matrix. It plays a fundamental role in regulating tumor initiation, progression, metastasis, and therapeutic response. Increasing evidence suggests that alterations in redox signaling, cytokine expression, metabolic adaptation, and intercellular communication within the TME profoundly shape cancer behavior and determine treatment outcomes. Understanding the molecular mechanisms that govern TME-mediated regulation of cancer stemness, apoptosis, and immune modulation is therefore critical for developing novel therapeutic strategies [1].

Emerging research emphasizes the metabolic, signaling heterogeneity, and cancer stemness driven by the TME. In oral cancer, epithelial cell adhesion molecule (EpCAM) signaling regulates cancer stem cell (CSC) traits, metastasis, and epithelial-to-mesenchymal transition (EMT) via pathways such as those involving Wnt/β-catenin and EGFR [2]. Recent studies have also highlighted the chaperone protein, glucose-regulated protein 78 (GRP78), and antioxidant signaling pathways influencing tumor biology through TME modulation [3,4,5]. Isoliquiritigenin (ISL), a bioactive flavonoid derived from licorice, has been investigated for its effects on gastric cancer stemness. ISL suppresses GRP78-mediated stemness, modulates the TME, and enhances chemosensitivity [6]. The ω-3 polyunsaturated fatty acid linolenic acid (LA) has been shown to inhibit gastric cancer stemness and promote apoptosis by downregulating nuclear factor erythroid 2-related factor 2 (Nrf2) expression. As Nrf2 is a master regulator of antioxidant defense and redox homeostasis, its suppression by LA enhances oxidative stress and caspase-3-mediated apoptosis, providing a promising nutritional approach for targeting cancer stem cells with minimal toxicity [7]. These findings emphasize the therapeutic potential of dietary components in restoring oxidative balance and disrupting tumor-supportive microenvironments.

Within the TME, hypoxia acts as a central regulator that shapes immune cell dynamics and modulates therapeutic responsiveness. The bidirectional interplay between cancer stemness and the hypoxic TME is a critical driver of tumor progression [8]. In non-small cell lung cancer, the interaction between hypoxia-inducible factor 1-alpha (HIF-1α) and programmed death-ligand 1 (PD-L1) expression has been linked to KRAS mutations, indicating that hypoxia-mediated signaling facilitates immune evasion and may serve as a predictive marker for immunotherapy response [9]. CSCs further exacerbate immune escape, therapeutic resistance, and tumor relapse by secreting immunosuppressive cytokines, recruiting regulatory immune cells, and reprogramming anti-tumor components within the TME, thereby establishing an immune-tolerant niche conducive to tumor survival and expansion [1]. Consequently, targeting the pivotal molecular networks that govern hypoxia and stemness holds significant potential for the development of innovative therapeutic strategies aimed at overcoming tumor resistance [10].

Collectively, these studies underscore the intricate and multifactorial nature of tumor microenvironmental regulation. From redox imbalance to hypoxia-driven immune modulation, the TME dictates cancer cell plasticity, survival, and therapeutic response. Integrative modulation of the TME, through nutritional intervention, redox balance regulation, cancer stemness regulation, and immune activation, offers a promising strategy to overcome drug resistance and enhance therapeutic efficacy across various cancer types. A deeper mechanistic understanding of TME-mediated signaling networks will pave the way for more effective, personalized therapeutic strategies in oncology.
